# Test–Retest Reliability of Mismatch Negativity (MMN) to Emotional Voices

**DOI:** 10.3389/fnhum.2018.00453

**Published:** 2018-11-15

**Authors:** Chenyi Chen, Chia-Wen Chan, Yawei Cheng

**Affiliations:** ^1^Department of Physical Medicine and Rehabilitation, National Yang-Ming University Hospital, Yilan, Taiwan; ^2^Graduate Institute of Injury Prevention and Control, Taipei Medical University, Taipei, Taiwan; ^3^Institute of Humanities in Medicine, Taipei Medical University, Taipei, Taiwan; ^4^Research Center of Brain and Consciousness, Shuang Ho Hospital, Taipei Medical University, Taipei, Taiwan; ^5^Institute of Neuroscience and Brain Research Center, National Yang-Ming University, Taipei, Taiwan; ^6^Department of Research and Education, Taipei City Hospital, Taipei, Taiwan

**Keywords:** emotional voice, mismatch negativity, test–retest reliability, attention disposition, circadian sessions

## Abstract

A voice from kin species conveys indispensable social and affective signals with uniquely phylogenetic and ontogenetic standpoints. However, the neural underpinning of emotional voices, beyond low-level acoustic features, activates a processing chain that proceeds from the auditory pathway to the brain structures implicated in cognition and emotion. By using a passive auditory oddball paradigm, which employs emotional voices, this study investigates the test–retest reliability of emotional mismatch negativity (MMN), indicating that the deviants of positively (happily)- and negatively (angrily)-spoken syllables, as compared to neutral standards, can trigger MMN as a response to an automatic discrimination of emotional salience. The neurophysiological estimates of MMN to positive and negative deviants appear to be highly reproducible, irrespective of the subject’s attentional disposition: whether the subjects are set to a condition that involves watching a silent movie or do a working memory task. Specifically, negativity bias is evinced as threatening, relative to positive vocalizations, consistently inducing larger MMN amplitudes, regardless of the day and the time of a day. The present findings provide evidence to support the fact that emotional MMN offers a stable platform to detect subtle changes in current emotional shifts.

## Introduction

Mismatch Negativity (MMN) is a differential wave obtained by subtracting the auditory event-related potential (ERP) component evoked by frequent, repetitive “standard” sounds from the ERP component evoked in response to the infrequent deviants that are interspersed within a constant auditory stream. It is defined as a negative ERP displacement, particularly, at the frontocentral and central scalp electrodes relative to a mastoid or nose reference electrode (Naatanen et al., 2007). The MMN reflects the early saliency detection of auditory stimuli that is generated in a hierarchical network involving primary and secondary auditory regions as well as specific brain regions regarding stimulus discrimination, based on the perceptual processes of physical features (Rinne et al., 2000; Doeller et al., 2003; Pulvermuller and Shtyrov, 2006; Garrido et al., 2008; Thonnessen et al., 2010). The “model-adjustment hypothesis“ of MMN indicates that the MMN is evinced from the comparison process between a sensory input and a “memory-based“ perceptual model (Naatanen and Winkler, 1999; Naatanen et al., 2005).

In the same vein, previous studies suggested that, in addition to many basic features of sounds such as frequency, duration, intensity, or even sound omissions, the MMN could also be utilized as an index of the salience of emotional voice processing (Schirmer and Kotz, 2006; Schirmer et al., 2008; Schirmer and Escoffier, 2010; Thonnessen et al., 2010). When meaningless syllables “dada” are spoken with emotionally neutral, happy, or disgusted prosodies, administered by way of a passive oddball paradigm, disgusted deviants in comparison with happy deviants have been seen to elicit stronger magnetoencephalographic counterparts of MMN (MMNm) and MMNm-related cortical activities in the right anterior insular cortex, a region that has been previously demonstrated as critical in the processing of negative emotions, such as disgusted facial expressions (Chen et al., 2014). This procedure has also been employed to measure voice and emotional processing in infants (Cheng et al., 2012; Zhang et al., 2014). Newborns have been found to be sensitive to emotional voices beyond specific language and exhibit the comparable ability with adults to process the affective information in voices (Fan et al., 2013; Hung and Cheng, 2014; Chen et al., 2015). Voices with affective information are supposed to elicit higher recruitment of associated resources than those with non-affective information.

Given the fact that MMN can be generated without the need of the subjects’ explicit attention toward the sound stimuli, it proves to be beneficial in investigations regarding populations with potential attention deficit comorbidities. It is noteworthy that even though attention is not necessary to evoke MMN, it has been observed that MMN is sensitive to attention. When the subject’s attention is directed elsewhere, the MMN is usually elicited quite similarly to when the sequence of standard and deviant sounds is attended. Nevertheless, MMN amplitude may be somewhat attenuated under certain conditions with highly focused attention elsewhere (Naatanen et al., 2007). In accordance with the most important implication of MMN serving as a valid tool to detect and assess neurological, neuropsychiatric, and neurodevelopmental disorders (Naatanen et al., 2015), as well as healthy aging (Naatanen et al., 2012), emotional MMN has also been linked with potential clinical ERP biomarkers, especially in detecting abnormalities in emotional processing, such as those observed in delinquents with conduct disorder symptoms, people with autism spectrum conditions, and positive symptoms in schizophrenia (Hung et al., 2013; Fan and Cheng, 2014; Chen et al., 2016a). Previous electroencephalography (EEG) studies have evaluated the reliability of MMN dependent on duration, frequency, and intensity changes in stimulus and provided its detectability and essentiality for potential ERP biomarkers in clinical research. Moderate to robust reliability for ERPs, ranging between 0.37 and 0.87, have been identified (Frodl-Bauch et al., 1997; Kathmann et al., 1999; Tervaniemi et al., 1999; Schroger et al., 2000; Light and Braff, 2005; Hall et al., 2006; Light et al., 2012). The replicability of MMNm has also been assessed, with reports stating high intraclass correlation coefficients (ICC) for duration (0.89), frequency (0.86), and omission (0.63∼0.9) deviants (Tervaniemi et al., 2005; Recasens and Uhlhaas, 2017).

It is noteworthy that while anxious states were purposely elicited in healthy participants by exposure to unpredictable aversive shocks, threat-induced anxiety was observed to induce anxious hypervigilance and produce an enlargement of the MMNm to pure tone deviants (Cornwell et al., 2017). Considering the emotional salience inherited in vocal expression, emotional MMN can help in identifying the comprehension of vocal emotionality as a special domain, beyond general processing and vigilance to mechanically stabilize environmental changes (Schirmer and Kotz, 2006). For instance, emotional MMN may serve as a proxy to access the ERP correlates of trait and state anxiety and the automatic emotional salience processing in the sleeping brain (Cheng et al., 2012; Chen et al., 2016b, 2017). While several studies have assessed the stability of MMN to non-vocal and pure tone deviants, test–retest reliability of emotional MMN is currently unclear. Given the use of emotional MMN as a potential proxy for approaching the automatic emotional saliency in individuals with aberrant emotional processing and attention deficit comorbidities, we evaluated the stability of emotional MMN under various attentional conditions as well as on different days and on different times of a day.

The test–retest reliability for different times of day was assessed by recording the ERPs in the mid-morning (10:30 AM) and in the mid-afternoon (15:30 PM) of the same day. Then, two weeks later, the same two sessions were recorded again, this time in order to examine the test–retest reliability of emotional MMN on different days. In order to assess the reliability under different attentional conditions, the participants were subjected to two different tasks (that were later used for comparison). In one task, they were required to direct their attention to a working memory task: first, they evaluated the position of an undergoing visual stimulus, and then they compared the position of the undergoing stimulus with that of the two trials before (*2*-back); in the other task, the subjects were asked to watch a silent movie.

For the end of this study, we hypothesize that the deviant stimuli embedded in the oddball paradigm (whether they are of positively or negatively spoken syllables), when compared to neutral standards, can evoke MMN as an outcome of the automatic discrimination of emotional salience. We further hypothesize that the neurophysiological estimates of MMN to positive and negative deviants will appear to be highly reproducible, irrespective of the attentional disposition generated in the participants by means of the tasks they are set to perform, namely watching a silent movie or engaging in a working memory task, thus providing evidence of emotional MMN as a stable platform to detect subtle changes in current emotional shifts.

## Materials and Methods

### Subjects

Twenty healthy volunteers (10 males), aged between 20 and 26 years, participated in this study after providing written informed consent and receiving monetary compensation for their participation. All participants had normal bilateral peripheral hearing (pure tone average thresholds <15 dB HL) and normal middle ear function at the time of testing. None of them had a history of neurological, endocrinal, and/or psychiatric disorders; no subjects were taking any medication at the time of testing. This study was approved by the ethics committee at National Yang-Ming University and conducted in accordance with the Declaration of Helsinki.

### Auditory Stimuli

**FIGURE 1:**
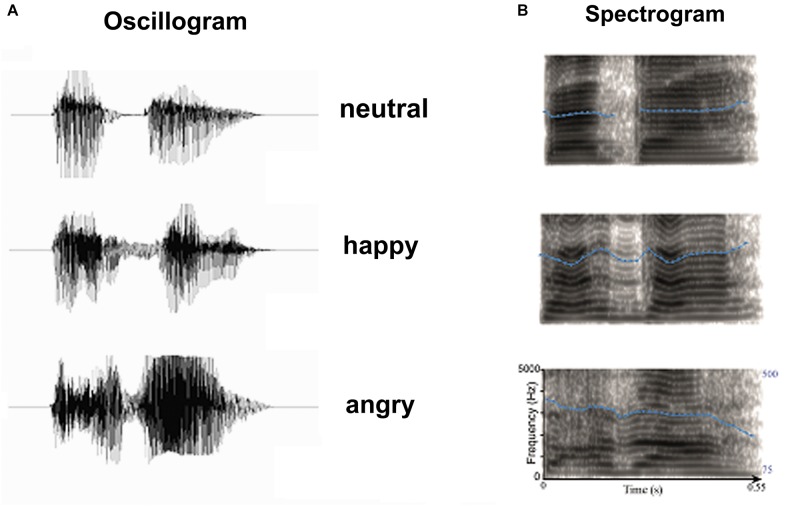
Acoustic properties of stimulus materials: **(A)** Oscillogram and **(B)** Spectrogram of auditory stimulus. Blue dot lines in the spectrum denote the flow of fundamental frequency at each time point, which represents the pitch flow of emotional syllables.

Three emotional syllables were used to elicit auditory ERPs. A female speaker from a performing arts school produced meaningless syllables “dada” with three sets of emotional (happy, angry, and neutral) prosodies (Cheng et al., 2012; Hung et al., 2013; Chen et al., 2014). Compared to fearful prosody, angry prosody showed more similarity in physical attribute with happy prosody (Supplementary Figure 3). On the other hand, both anger and happiness are approach-related affects, whereas fear reflects the opposing direction of motivational engagement (withdrawal or avoidance). Therefore, we selected happy voices to stand for the positive emotion and angry voices for the negative one. Emotional syllables were edited to become equally long (550-ms) and loud (min: 57 dB; max: 62 dB; mean 59 dB) using Cool Edit Pro 2.0 and Sound Forge 9.0 (Figure 1). Each syllable set was rated for emotionality on a 5-point Likert-scale by a total of 120 listeners (60 men). For the angry set, listeners classified each angry prosody on a scale of extremely angry to not angry at all. For the happy set, listeners classified each happy prosodies on a scale of extremely happy to not happy at all. For the neutral set, listeners classified the neutral prosodies on a scale of extremely emotional to not emotional at all. Emotional syllables that were consistently identified as extremely angry and extremely happy (i.e., the highest ratings), as well as the most emotionally neutral (i.e., the lowest rating), were used as the stimuli. The Likert-scale (mean ± SD) of happy, angry, and neutral syllables were 4.34 ± 0.65, 4.26 ± 0.85, and 2.47 ± 0.87, respectively. Neutral syllables were employed as standard (S), and happy and angry syllables designed as two isometric deviants (D1 and D2) followed the oddball paradigm. Each session consisted of 800 standards, 100 D1 s, and 100 D2 s. A minimum of two standards was presented between any two deviants. The successive deviants were always diverse. The stimulus onset asynchrony was 1200 ms.

### Procedures

The experiment consisted of four sessions. The first two sessions were recorded in the mid-morning (10:30 AM) and mid-afternoon (15:30 PM) to examine the test–retest reliability of emotional MMN at different times of the day. Then, two weeks later, the same two sessions were recorded again to examine the test–retest reliability of emotional MMN on different days. The procedures and the order of the experiments were identical on the two different days. Half of the participants (*n* = 10, 5 males) were asked to evaluate the position of a visual stimulus; then, they were asked to compare the position of an undergoing stimulus with that of the previous two trials (*2*-back), and during all these sessions, the auditory ERPs to irrelevant emotionally spoken syllables “dada” were being recorded. The other half of the subjects (*n* = 10, 5 males) watched a silent movie without paying attention to the sounds during the passive auditory oddball paradigm. The factor of attention on emotional MMN was employed by manipulating the task load on irrelevant working memory processing (*2*-back).

### *2*-Back Working Memory Task

The software for the *2*-back task, developed by Paul Hoskinson, comes from an online website (Brain Workshop^1^). In every trial, each blue square was displayed in a 3 × 3 matrix and the stimulus onset asynchrony was varied (max: 3 s). During the *2*-back task, participants were requested to decide whether the undergoing stimulus was at the same location as the stimulus of the previous two trials by pressing the “A” button on a keyboard. The subjects were able to practice the *2*-back task, immediately before the EEG recording, in order to get familiar with it beforehand.

### Electroencephalogram Apparatus and Recording

The EEG experiment was conducted in a sound-attenuated, dimly lit and electrically shielded room. Stimuli were presented binaurally via two loudspeakers placed at approximately 30 cm on the right and left sides of the subject’s head. The sound-pressure level (SPL) peaks of different types of stimuli were equalized to eliminate the effect of the substantially greater energy of the angry stimuli. The mean background noise level was around 35 dB SPL. The EEG was continuously recorded from 32 scalp sites using electrodes mounted on an elastic cap and positioned according to the modified International 10–20 system, with the addition of two mastoid electrodes. The electrode at the right mastoid (A2) was used as an online reference. Eye blinks and vertical eye movements were monitored with electrodes located above and below the left eye. The horizontal electrooculogram (EOG) was recorded from electrodes placed laterally 1.5 cm to the left and right external canthi. A ground electrode was placed on the forehead. Electrode/skin impedance was kept to < 5 kΩ. Channels were re-referenced off-line to the average of the left and right mastoid recordings [(A1 + A2)/2]. Signals were sampled at 500 Hz, band-pass filtered at 0.1–100 Hz, and epoched over an analysis time of 600 ms, including the prestimulus time of 100 ms used for baseline correction. An automatic artifact rejection system excluded from the average of all trials containing transients exceeding ± 70 μV at recording electrodes and ± 100 μV at the horizontal EOG channel. Furthermore, the quality of ERP traces was ensured by careful visual inspection in every subject and trial by applying appropriate digital, zero-phase shift band-pass filters (0.1–50 Hz, 24 dB/octave). Gratton-Coles ocular artifact correction was performed on the EEG data to identify and correct ocular movements, with seeded off electrode Fp1 (Gratton et al., 1983). The first ten epochs of each sequence were omitted from the averaging in order to exclude unexpected large responses elicited by the initiation of the sequences.

### Data Analysis

The amplitudes of emotional MMN were analyzed as an average within a 50-ms time window surrounding the peak latency at the electrode sites F3, Fz, F4, C3, Cz, and C4. The MMN peak was defined as the largest negativity in the subtraction between the deviant and standard ERPs, during a period of 150–250 ms after sound onset. Only the standards presented before the deviants were included in the analysis. To avoid the order effect that might carry over within a short range of interval in the same day, statistical analyses on emotional MMN were conducted separately for morning and afternoon period, using a four-way analysis of variance (ANOVA) comprising the group factor Attention (silent movie vs. *2*-back), and the repeated-measures factors Deviant Type (angry vs. happy), Session (day 1 vs. day 2), and Electrode (F3, Fz, F4, C3, Cz, and C4). The dependent variables were the mean amplitudes and peak latencies of the MMN at the selected electrode sites. Degrees of freedom were corrected using the Greenhouse-Geisser method. The *post hoc* comparison was conducted only when preceded by significant main effects. Statistical power (1 – β) was estimated by G^∗^Power 3.1 software (Faul et al., 2009).

Test–retest reliability was calculated and estimated by the values of the ICC (Shrout and Fleiss, 1979). The ICC could assess the degree of relative consistency among various measures across different sessions. It was calculated as the ratio between the between-subject variance and the total variance across all measures and participants. Unlike Pearson’s correlation coefficient, which measures the strength of the linear association between two measures, the ICC takes into account the variability of the total sample and reflects the agreement of the measures obtained across sessions. An ICC value of 1 indicates perfect within-subject reliability, whereas an ICC of 0 indicates no reliability. The ICC was assessed for both amplitude and latency signals between different days and circadian sessions. Step-down Holm–Bonferroni correction was used to control the familywise error to counteract the problem of multiple comparisons in all reported results (both the main manuscript and the supporting materials).

## Results

### MMN Amplitude and Peak Latency

**FIGURE 2:**
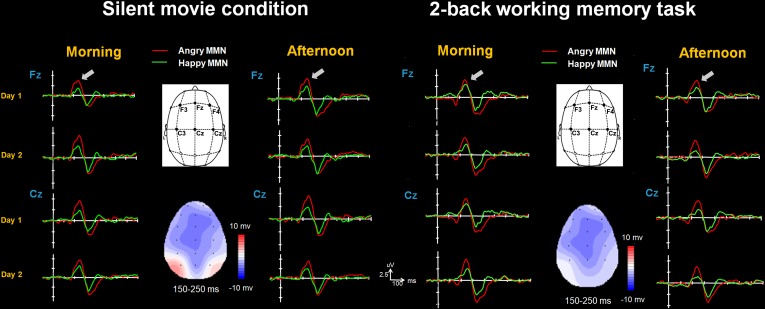
The happy MMN (green lines) and angry MMN (red lines) recorded from F3 to C4 electrodes, averaged across all subjects when watching the silent movie and performing the *2*-back working memory task. The gray arrows indicate the time window of MMN.

**FIGURE 3:**
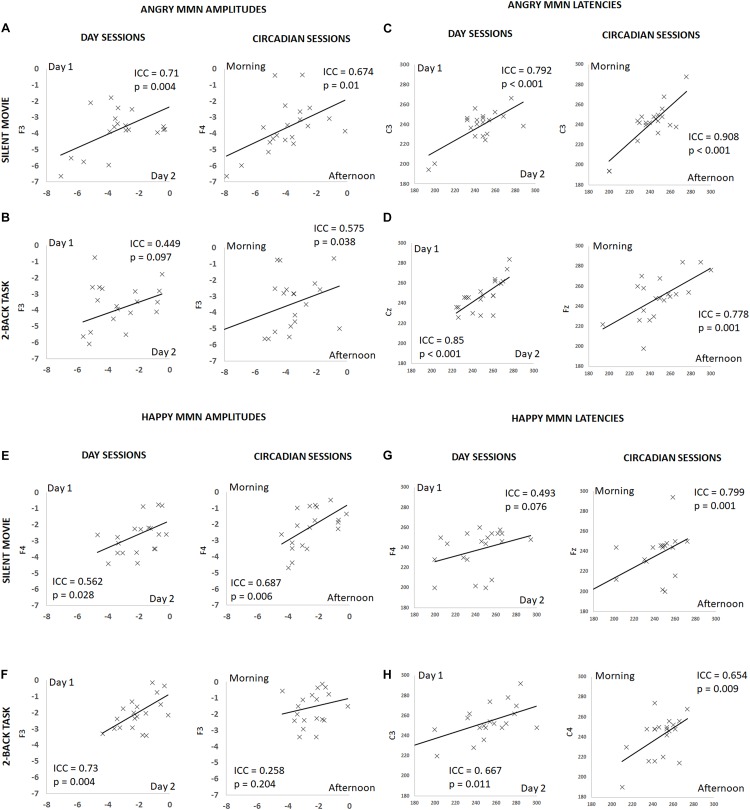
Summary of individual estimates for intraclass correlation coefficient (ICC) in amplitude and latency, for angry and happy MMN, on different days and at different times of a day, during the silent movie and *2*-back working memory task conditions. *X*-axis denotes individual values in day 2 or afternoon session and *Y*-axis in day 1 or morning session. Plots show test–retest angry MMN amplitudes in the silent movie condition **(A)** and in the *2*-back condition **(B)**, test–retest angry MMN peak latencies in the silent movie condition **(C)** and in the *2*-back condition **(D)**, test–retest happy MMN amplitudes in the silent movie condition **(E)** and in the *2*-back condition **(F)**, and test–retest happy MMN peak latencies in the silent movie condition **(G)** and in the *2*-back condition **(H)**.

In general, morphology and amplitudes of the grand average curves are quite similar in the morning and afternoon periods of day 1 and day 2. This was confirmed by the statistical analysis conducted on MMN amplitudes (Figures 2, 3 and Supplementary Figures 1, 2). In the morning period, none of the Session effect was found either as a main effect [*F*(1,18) = 1.88, *p* = 0.19, η^2^ = 0.094, (1-β) = 13%] or when interacting with other variables (all *p* > 0.2). This was also true for the afternoon period [main effect of Session: *F*(1,18) = 1.13, *p* = 0.3, η^2^ = 0.059, (1-β) = 6%; interactions between Session and other variables: all *p* > 0.2]. Both morning and afternoon periods showed main effects of the Deviant Type [morning: *F*(1,18) = 30.44, *p* < 0.001, η^2^ = 0.628, (1-β) = 96%; afternoon: *F*(1,18) = 46.4, *p* < 0.001, η^2^ = 0.72, (1-β) = 99%] and the Electrode [morning: *F*(5,90) = 4.48, *p* = 0.01, η^2^ = 0.199, (1-β) = 33%; afternoon: *F*(5,90) = 2.83, *p* = 0.02, η^2^ = 0.136, (1-β) = 16%]. Angry MMN (morning: 3.99 ± 0.28; afternoon: 3.39 ± 0.25) was significantly stronger than happy MMN (morning: 2.35 ± 0.16; afternoon: 1.82 ± 0.18) irrespective of the session or the attention. The MMN had the strongest amplitudes at electrode Fz (morning: 3.49 ± 0.22, afternoon: 2.77 ± 0.26) and Cz (morning: 3.43 ± 0.19, afternoon: 2.74 ± 0.18) as compared to F3 (morning: 2.97 ± 0.25, afternoon: 2.52 ± 0.22), F4 (morning: 3.18 ± 0.21, afternoon: 2.74 ± 0.22), C3 (morning: 2.98 ± 0.18, afternoon: 2.43 ± 0.17), or C4 (morning: 2.97 ± 0.17, afternoon: 2.44 ± 0.16) irrespective of the session or the attention [Fz vs. F3: mean difference -0.387, *p* < 0.001; Fz vs. C3: mean difference -0.426, *p* = 0.021; Fz vs. C4: mean difference -0.427, *p* = 0.001; Cz vs. F3: mean difference -0.339, *p* = 0.015; Cz vs. C3: mean difference -0.378, *p* = 0.001; Cz vs. C4: mean difference -0.379, *p* < 0.001]. Attention did not affect emotional MMN amplitudes, as shown by testing the group factor and its interaction with the repeated measures of Session, Deviant Type, and Electrodes factors. Neither a main effect was found [morning: *F*(1,18) = 0.4, *p* = 0.54, η^2^ = 0.022, (1-β) = 5%; afternoon: *F*(1,18) = 0.5, *p* = 0.49, η^2^ = 0.027, (1-β) = 5%] nor was there any significant interaction [Attention × Session/morning: *F*(1,18) = 0.22, *p* = 0.65, η^2^ = 0.012, (1-β) = 5%; Attention × Session/afternoon: *F*(1,18) = 1.19, *p* = 0.29, η^2^ = 0.062, (1-β) = 6%; Attention × Deviant Type/morning: *F*(1,18) = 0.7, *p* = 0.41, η^2^ = 0.037, (1-β) = 5%; afternoon: *F*(1,18) = 1.59, *p* = 0.22, η^2^ = 0.081, (1-β) = 7%; Attention × Session × Deviant Type × Electrode/morning: *F*(5,90) = 1.61, *p* = 0.22, η^2^ = 0.082, (1-β) = 13%; Attention × Session × Deviant Type × Electrode/afternoon: *F*(5,90) = 1.22, *p* = 0.31, η^2^ = 0.063, (1-β) = 9%].

The repeated-measures ANOVA conducted on the MMN peak latencies also revealed that none of Session effect was found either as a main effect [morning: *F*(1,18) = 1.55, *p* = 0.23, η^2^ = 0.079, (1-β) = 10%; afternoon: *F*(1,18) = 1.86, *p* = 0.19, η^2^ = 0.094, (1-β) = 13%] or interacting with other variables (all *p* > 0.3). Attention did not affect the emotional MMN peak latencies, as shown by testing the group factor and its interaction with the Session, Deviant Type, and Electrodes factors. Neither a main effect was not found [morning: *F*(1,18) = 2.27, *p* = 0.15, η^2^ = 0.112, (1-β) = 9%; afternoon: *F*(1,18) = 2.41, *p* = 0.14, η^2^ = 0.118, (1-β) = 9%] nor was there any significant interaction [Attention × Session/morning: *F*(1,18) = 0.94, *p* = 0.35, η^2^ = 0.049, (1-β) = 6%; Attention × Session/afternoon: *F*(1,18) = 0.03, *p* = 0.86, η^2^ = 0.002, (1-β) = 5%; Attention × Deviant Type/morning: *F*(1,18) = 0.005, *p* = 0.94, η^2^ < 0.001, (1-β) < 5%; Attention × Deviant Type/afternoon: *F*(1,18) = 0.005, *p* = 0.94, η^2^ < 0.001, (1-β) < 5%; Attention × Session × Deviant Type × Electrode/morning: *F*(5,90) = 0.56, *p* = 0.73, η^2^ = 0.03, (1-β) = 6%; Attention × Session × Deviant Type × Electrode/afternoon: *F*(5,90) = 0.43, *p* = 0.69, η^2^ = 0.023, (1-β) = 6%].

### Test–Retest Reliability

The ICC for angry MMN amplitudes were robust on different days and at different times of a day (Circadian: morning vs. afternoon) during the silent movie condition (Day: ICC = 0.71, *p* = 0.004; Circadian: ICC = 0.674, *p* = 0.01) (Figure 3A and Table 1). In the *2*-back working memory condition, the ICC for angry MMN amplitudes showed moderate reliability on different days and at different times of a day (Day: ICC = 0.449, *p* = 0.097; Circadian: ICC = 0.575, *p* = 0.038) (Figure 3B and Table 1). The ICC indicated overall strong values for the angry MMN peak latencies, both in the silent movie (Day: ICC = 0.792, *p* < 0.001; Circadian: ICC = 0.908, *p* < 0.001) and *2*-back conditions (Day: ICC = 0.85, *p* < 0.001; Circadian: ICC = 0.778, *p* = 0.001) (Figures 3C,D and Table 1).

**Table 1:** Test–retest intraclass correlation coefficients (ICC) for emotional MMN amplitudes and latencies, separately for angry- and happy-syllable deviants, in each group watching the silent movie (*n* = 10) or performing the *2*-back working memory task (*n* = 10), on different days (day 1 vs. day 2) and at different times of the day (Circadian: morning vs. afternoon).

Electrode	Happy MMN amplitude				Angry MMN amplitude	
	Day	Circadian			Day	Circadian
Silent movie					
F3	0.465	0.56			0.71**	0.397
Fz	0.34	0.39			0.216	–0.343
F4	0.562	0.687**			0.57	0.674**
C3	0.163	0.058			0.591	0.332
Cz	0.444	0.191			0.554	0.319
C4	0.452	0.573			0.321	0.358
*2*-back working memory task					
F3	0.73**	0.258			0.449	0.575
Fz	0.421	0.101			0.268	0.555
F4	0.565	0.032			0.06	0.543
C3	0.62	0.178			0.375	0.418
Cz	0.377	–0.028			0.156	0.484
C4	0.512	0.091			0.152	0.326
	**Happy MMN peak latency**				**Angry MMN peak latency**	
Silent movie					
F3	0.477	0.762***			0.649*	0.448
Fz	0.41	0.799***			0.29	0.394
F4	0.493	0.257			0.722**	0.798***
C3	0.075	0.661**			0.792***	0.908***
Cz	0.311	0.337			0.712**	0.795***
C4	0.458	0.214			0.705**	0.689**
*2*-back working memory					
F3	0.483	0.499			0.593	0.645*
Fz	0.558	0.594			0.768***	0.778***
F4	0.272	0.458			0.697**	0.751**
C3	0.667	0.638			0.47	0.726**
Cz	–0.075	0.424			0.85***	0.594
C4	0.534	0.654			0.255	0.556
	

*^∗^P < 0.05; ^∗∗^*P* < 0.01; ^∗∗∗^*P* < 0.001; corrected for multiple comparisons.*

Similarly, the ICC values for happy MMN amplitudes indicated fair to good reliability on different days and at different times of a day during the silent movie condition (Day: ICC = 0.562, *p* = 0.028; Circadian: ICC = 0.687, *p* = 0.006) (Figure 3E and Table 1). The ICC for happy MMN amplitudes in the *2*-back condition evidenced a good reliability on different days, but a weak reliability at different times of a day (Day: ICC = 0.73, *p* = 0.004; Circadian: ICC = 0.258, *p* = 0.204) (Figure 3F and Table 1). Happy MMN peak latencies obtained during silent movie (Day: ICC = 0.493, *p* = 0.076; Circadian: ICC = 0.799, *p* = 0.001) and 2-back conditions (Day: ICC = 0.667; *p* = 0.011; Circadian: ICC = 0.654, *p* = 0.009) showed less robust ICC values when compared to its values for angry MMN peak latencies (Figures 3G,H and Table 1).

Fisher r-to-z transformation was conducted on the highest ICC values of each category to examine whether there were significant ICC differences between amplitudes and latencies, *2*-back memory tasks and silent movie condition, as well as happy and angry emotion. The ICC of angry MMN was higher than that of happy MMN in both the silent movie (Day: ICC angry MMN latency = 0.792 vs. ICC happy MMN latency = 0.075, *p* = 0.004; Circadian: ICC angry MMN latency = 0.908 vs. ICC happy MMN latency = 0.661, *p* = 0.04) and the working memory condition (Day: ICC angry MMN latency = 0.85 vs. ICC happy MMN latency = -0.075, *p* < 0.001). The ICC for latency was higher than the ICC for amplitude in both happy (Circadian: ICC amplitude = 0.39 vs. ICC latency = 0.799, *p* = 0.046) and angry MMN (Day: ICC amplitude = 0.156 vs. ICC latency = 0.85, *p* = 0.001; Circadian: ICC amplitude = 0.332 vs. ICC latency = 0.908, *p* < 0.001). The ICC in the silent movie condition was found to be higher than that of in the working memory condition (Happy MMN amplitudes: ICC movie = 0.687 vs. ICC memory = 0.032, *p* = 0.018).

## Discussion

In this study, we investigated the test–retest reliability of emotional MMN that was elicited by vocal emotional expressions. The results demonstrated that both deviants of positively (happily)- and negatively (angrily)-spoken syllables when compared with neutral standards could reliably trigger MMN in response to emotional salience processing. Specifically, the neurophysiological estimates of MMN to both angry and happy deviants appeared to be highly reproducible, irrespective of whether a passive auditory oddball paradigm was incorporated during the silent movie observation or a working memory performance task (Table 1).

Emotional MMN amplitudes and latencies were neither significantly affected by the different sessions (day 1 vs. day 2) and the attentional loadings (silent movie vs. *2*-back task) nor by any other significant interactions between these two variables in both morning and afternoon sessions, respectively. In accordance with our previous findings, angry MMN elicited stronger amplitudes than happy MMN (Fan et al., 2013; Fan and Cheng, 2014; Chen et al., 2016a). Angry MMN, rather than neutral or non-vocal MMN amplitudes, was associated with the severity of autistic traits, indicating atypical emotional voice processing at the early automatic stage (Fan and Cheng, 2014). Higher state anxiety and ensuing heart rate acceleration was also found to be associated with larger angry MMN amplitudes but not neutral MMN amplitudes (Schirmer and Escoffier, 2010). Acute testosterone administration modulated neural dynamics of voice perception and emotional MMN, but not of non-vocal MMN, indicating the role o neural dynamics on modulating pre-attentive sensory processing and involuntary attention switches in response to emotional voices (Chen et al., 2015). Taken together, voice- and emotion-dependent modulation specifically found in vocal MMN, rather than non-vocal MMN, is difficult to synthesize with bottom-up neural adaptation or oscillatory rebound accounts (May and Tiitinen, 2010). Thus, it may instead support the involvement of predictive top-down mechanisms and memory-based model-adjustment hypothesis (Naatanen et al., 2005; Garrido et al., 2008; Wacongne et al., 2012; Lieder et al., 2013). With a five-way ANOVA comprising the group factor Attention (silent movie vs. 2-back), and the repeated-measures factors Deviant Type (angry vs. happy), Session (day 1 vs. day 2), Electrode (F3, Fz, F4, C3, Cz, and C4), and an additional variable of Time (morning vs. afternoon), we identified a main effect of Time besides the effect uncovered in the main text (Supplementary Results). The MMN had larger amplitudes in the morning (3.17 ± 0.177) compared to afternoon session (2.62 ± 0.18) as a manifesto of practice/order effect in which the subjects do exactly the same tasks within hours.

Angry MMN amplitudes and latencies showed robust ICC test–retest reliability, whereas happy MMN amplitudes and latencies showed fair to good ICC values, over day and circadian sessions during the silent movie condition. Negative emotionality, which has a greater effect on one’s psychological state and processes than neutral or positive ones, have been reported and largely converged with factors previously shown to impact the processing of emotional facial expressions, unpleasant thoughts, or social interactions, suggesting a modality-independent impact of negativity bias (Kanouse and Hanson, 1972; Baumeister et al., 2001; Rozin and Royzman, 2001; Young et al., 2017). Threatening voices, such as angry vocalizations that could consistently trigger an alert response across different environmental conditions, served as the origin for evolving a specialized signal processing that facilitates survival.

During the silent movie condition, the ICC for both angry and happy MMN amplitudes indicated robust and fair reliabilities on different days and at different times of a day. However, during the *2*-back working memory task condition, the ICC values showed moderate reliabilities for angry MMN amplitudes and weak reliabilities for happy MMN amplitudes (Figure 3B and Table 1). Emotional processing is closely intertwined with the attention control system, such that emotionally salient and threatening stimuli will automatically capture attention (Taylor and Fragopanagos, 2005; Carlson et al., 2009; Yamaguchi and Onoda, 2012). Meanwhile, diverting attention is also found to suppress emotional influences in human amygdala responses (Morawetz et al., 2010; Pourtois et al., 2013). While the neural processing of emotional voices, beyond low-level acoustic features, recruits a processing chain that proceeds from the auditory pathway to brain structures implicated in social cognition, the heavy demand for cognitive resources and high load of task-irrelevant streams result in reduced emotional MMN amplitudes during the *2*-back working memory task (Schirmer and Kotz, 2006; Fan et al., 2013). Given that we have neither found a significant main effect nor any other interaction of attention, the high load of cognitive demand does not affect emotional MMN amplitudes in this cohort. The neural response to emotional salience that varied with high competition between automatic emotional processing and task-irrelevant loading may possibly manifest the fact that emotional MMN is sensitive and prone to capture the subtle individual differences in trait anxiety, as well as current emotional states, where present anxiety shifts from current vigilance to threatening signals and overcomes the competition of cognitive resources (Schirmer and Escoffier, 2010; Chen et al., 2015, 2017). Despite the fact that we did not find significant differences in emotional MMN during the *2*-back working memory task and the silent movie conditions, the limitation of the small group size and the variance in attentional load (e.g., n-back) should be taken into account (Fan et al., 2013). Future studies to examine task difficulty and test–retest reliability of (emotional) MMN are warranted.

Notably, when extremely high anxiety was deliberately induced by an unpredictable electrical shock, the enlarged pure-tone-MMNm was found to reflect an anxious hypervigilance state. Importantly, this heightened neurophysiological index of anxious hypervigilance could be reversed by an inhibitory gamma-aminobutyric acidergic action with alprazolam (Cornwell et al., 2017). Given the fact that voice processing the sounds of kin species has its unique phylogenetic and ontogenetic significance, an oddball paradigm deployed with emotional voices offers a platform to detect the subtle changes in current emotional shifts (Belin et al., 2000; Belin and Grosbras, 2010; Schirmer and Escoffier, 2010).

Our findings suggest that emotional MMN responses could be reliably obtained in conditions without task demands. However, whether the variation of test–retest emotional MMN in the individual level reflects a moment-to-moment emotional state shift remains as an important and promising inquiry. Our study underscores and sheds light on the need to take into account the instant, current emotional state at the individual level while trying to utilize MMN as a biomarker for diagnosis and translational medicine.

## Author Contributions

YC designed the study. CC and C-WC organized the database and performed the statistical analysis. CC wrote the first draft of the manuscript. CC, C-WC, and YC wrote sections of the manuscript. All authors contributed to manuscript revision, read, and approved the submitted version.

## Conflict of Interest Statement

The authors declare that the research was conducted in the absence of any commercial or financial relationships that could be construed as a potential conflict of interest.

## Footnotes

1 http://brainworkshop.sourceforge.net/
