# Supplementary material for: Test–Retest Reliability of Mismatch Negativity (MMN) to Emotional Voices
Source: Front Hum Neurosci. 2018 Nov 15;12:453. doi: 10.3389/fnhum.2018.00453 (PMC6249375; doi:10.3389/fnhum.2018.00453)
Supplement: Supplementary file 1 [file Data_Sheet_1.PDF]

SUPPLEMENTARY MATERIALS

Supplementary Figure 1: ERPs of neutral-syllable standards (black bold lines), happy-syllable and angry-syllable deviants (gray bold lines and black think lines), recorded from F3 to C4 electrodes, averaged across all subjects and all sessions when watching the silent movie. The transparent gray rectangles indicate the time window of MMN.

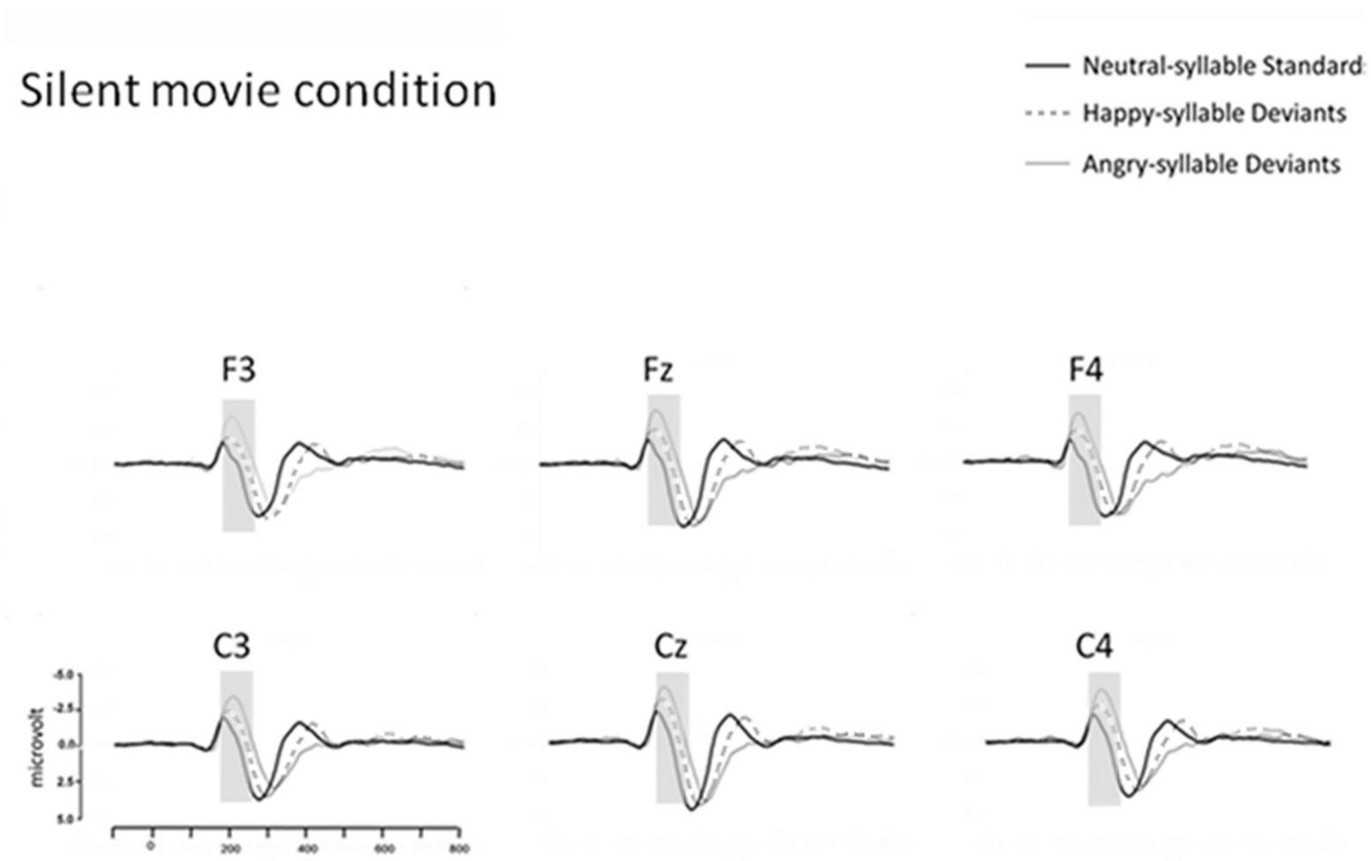

Supplementary Figure 2: ERPs of neutral-syllable standards (black bold lines), happy-syllable and angry-syllable deviants (gray bold lines and black think lines), recorded from F3 to C4 electrodes, averaged across all subjects and all sessions when performing the *2*-back working memory task. The transparent gray rectangles indicate the time window of MMN.

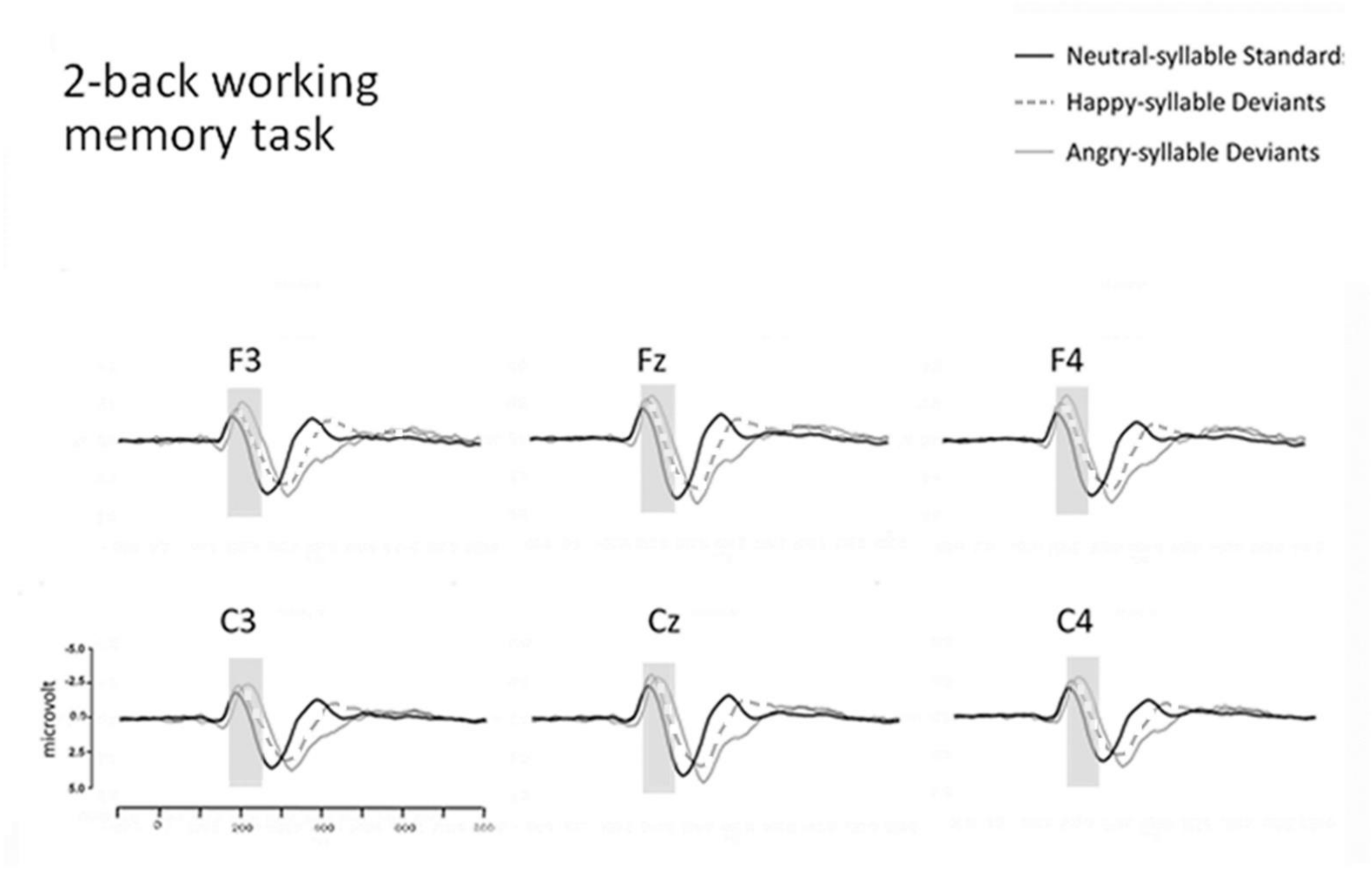

Supplementary Figure 3: Acoustic properties of stimulus materials.

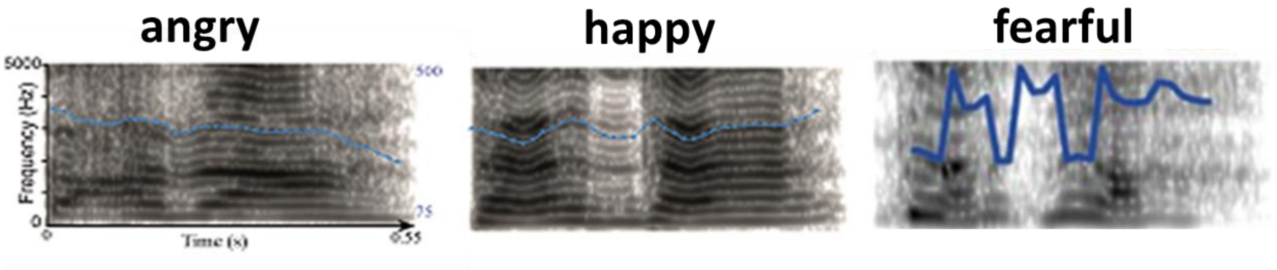

Supplementary Results:

A five-way ANOVA comprising the group factor Attention (silent movie vs. *2*-back), and the repeated-measures factors Deviant Type (angry vs. happy), Session (day 1 vs. day 2), Time (morning vs. afternoon), and Electrode (F3, Fz, F4, C3, Cz, C4) was conducted to examine whether morning/afternoon results in the session effect.

Besides the effect uncovered in the main text, the main effect of Time reached significance [F (1, 18) = 10.98, p = 0.19, η^2 = 0.38]. MMN has larger amplitudes in the morning (3.17 ± 0.177) compared to afternoon session (2.62 ± 0.18). This could be partially attributed to the habituation or order effect that

carried over within a short range of interval in the same day. None of interactions between Time and other variables reached significance level. The main effect of Deviant Type [F (1, 18) = 47.71, $p < 0.001$, $\eta^2 = 0.73$] and Electrode [F (5, 90) = 5.25, $p = 0.009$, $\eta^2 = 0.22$] remained significant after inclusion of the extra variable Time. Session did not affect emotional MMN amplitudes, as none of its main effect and its interaction with other variables (Time, Deviant Type, Electrodes, and Attention). Attention did not affect emotional MMN amplitudes, as none of its main effect and its interaction with other repeated measure factors (Time, Session, Deviant Type, and Electrodes).
